# The Relationship Between Employment Equity Perceptions and Psychological Ownership in a South African Mining House: The Role of Ethnicity

**DOI:** 10.1007/s11205-015-0972-z

**Published:** 2015-05-07

**Authors:** Chantal Olckers, Llewellyn van Zyl

**Affiliations:** Department of Human Resource Management, University of Pretoria, Private Bag X20, Hatfield, 0028 South Africa; Optentia Research Programme, Department of Industrial and Organisational Psychology, North-West University, Private Bag X1, Mahikeng, 1900 South Africa

**Keywords:** Employment equity, Psychological ownership, Structural equation modelling

## Abstract

Psychological ownership is a cognitive–affective construct based on individuals’ feelings of possessiveness towards and of being psychologically tied/attached to objects that are material (e.g. tools or work) and immaterial (e.g. ideas or workspace) in nature. Research suggests that psychological ownership could be influenced by various individual, organisational and contextual factors. The South African Employment Equity Act, which was implemented to grant equitable opportunities to previously disadvantaged employees, could be a significant contextual factor affecting psychological ownership, due to perceptions associated with inequality. Ethnicity may also act as a moderator for the relationship between perceptions of employment equity and psychological ownership. The objective of this study was to investigate the relationship between employment equity perceptions and psychological ownership and to explore whether ethnicity plays a moderating role in the relationship. A cross-sectional survey design was employed with a purposeful sample of 202 respondents employed in a large South African mining house. Pearson product–moment correlations and structural equation modelling confirmed that employment equity perceptions could predict the five components of psychological ownership. However, the results revealed that ethnicity has no moderating effect on the relationship between perceptions of employment equity and the emergence of psychological ownership. By implication, organisations that seek to retain employees targeted through equity initiatives need to find ways to enhance and develop the psychological ownership of these employees. The research contributes new insights into and knowledge of how contextual factors could influence employees’ psychological ownership.

## Introduction

Research on psychological ownership in organisations has increased over the last decade (Olckers 2013). Psychological ownership refers to the psychological experience of an employee when he/she develops possessive feelings for and attachment to a variety of objects in the organisation (Pierce et al. 2001, 2003). Several studies have revealed that the presence of psychological ownership among organisational members leads to more positive work-related attitudes, such as job satisfaction and commitment (O’Driscoll et al. 2006; Olckers 2013; Mayhew et al. 2007; VandeWalle et al. 1995), and behaviours such as organisational citizenship behaviours, extra-role behaviour and intentions to stay in the organisation (Avey et al. 2009; Olckers 2013; Van Dyne and Pierce 2004; Wagner et al. 2003).

However, according to Pierce et al. (2001) several factors could influence the emergence of psychological ownership, one of which pertains to contextual factors. In South Africa, a significant contextual factor in the form of government legislation is the Employment Equity Act. The Employment Equity Act (Act No 55 of 1998) was implemented in South Africa to achieve equitable opportunities for previously disadvantaged employees in order to rectify past injustices. However, research shows that individuals of various ethnic groups in South Africa have mixed feelings towards the implementation of the Act (Booysen 2007; Booysen and Nkomo 2014; Holtzhausen 2008). White individuals believe that the Act discriminates against minorities, whereas African individuals believe that it is retribution for suffering in the past (Holtzhausen 2008). As a result, ethnicity could significantly impact on the relationship between perceptions associated with employment equity and psychological ownership.

The aim of the study was therefore to investigate the relationship between employment equity perceptions and psychological ownership, and to explore the moderating role of ethnicity on this relationship.

## Literature Review

### Psychological Ownership Defined

Psychological ownership is defined by Pierce et al. (2001, 2003) as a state of mind, a feeling that a person has ownership over something or a target, even in the absence of legal ownership. Psychological ownership can be directed at a variety of objects/targets, including an organisation, a job, or a work project, and is considered to be a sense of possession of an object whereby the object becomes an extension of the self and is closely linked to the individual’s identity and has affective and cognitive elements (Pierce et al. 2001).

### The Motives of Psychological Ownership

Psychological ownership exists because it satisfies both generic and socially generated motives of individual human beings: a need for *efficacy and effectance, self*-*identity,* and *having a place* (Pierce et al. 2001, 2003; Van Dyne and Pierce 2004). More specifically psychological ownership aims to satisfy the need to be efficacious and in control of one’s environment in order to produce self-perceived desirable life/work outcomes (self-efficacy; Furby 1978; Olckers 2013), to define and express self-identity to others (self-identity; Dittmar 1992) and to have a place, a certain own area in which to safely and continually satisfy the other two motives (belongingness; Porteous 1976). Individuals can therefore develop feelings of ownership over a variety of objects as long as these objects allow the motives in question to operate and be satisfied.

Building on the three recognized dimensions described by Pierce et al. (2001) of self-efficacy, self-identity and belongingness (having a place), Avey et al. (2009) posited *accountability* and *territoriality* as additional dimensions of psychological ownership. Accountability refers to the tendency of an individual to feel responsible and to hold other individuals as well as their organisation accountable for the targets of ownership. Territoriality refers to the tendency of individuals to protect or defend any influence over their object of ownership. Avey et al. (2009) further distinguished between two forms of psychological ownership, namely *preventive* and *promotive* psychological ownership. A preventive focus is a concern to avoid punishment by keeping to rules and obligations. Territoriality forms part of this focus whereas self-efficacy, self-identity, belongingness and accountability are seen as promotive forms of psychological ownership. A promotive focus is concerned with what to do to fulfil the hopes and aspirations of an individual.

### The Paths to Psychological Ownership

According to Pierce and his colleagues (Pierce et al. 2001, 2003; Van Dyne and Pierce 2004) there are three major routes or paths by which feelings of ownership for a particular object emerge: (a) control over the target, (b) intimate knowledge of the target and (c) investment of the self in the target. In other words, the ability to use and control objects, through association and familiarity with them, and by investment of individual energy, time, effort, and attention in them, leads to ownership feelings and the union of the self with the object (Olckers 2013). Thus, when employees exercise greater amounts of control, intimately come to know, and invest themselves in the target of ownership, a sense of responsibility takes root and possessive feelings develop.

### Factors Influencing Psychological Ownership

According to Pierce et al. (2001), the emergence of psychological ownership can be influenced by a number of factors, such as target, individual, process and contextual factors. For the purposes of this study, the focus will be on contextual factors and specifically on their structural elements.

Structural elements of the context or ‘fences’, such as norms, rules, laws and hierarchy, may prevent individuals from developing feelings of ownership. These structural aspects that are ‘placed’ around objects stand between an individual and that individual’s potential target of ownership and might block the fulfilment of one or more of the motives of ownership by ‘fencing in’ the object (Pierce et al. 2001). In the South African contest, one such ‘fence’ could be associated with various forms of legislation such as the Employment Equity Act.

### Employment Equity Act

A significant contextual factor in the South African context in the form of government legislation is the Employment Equity Act. The Employment Equity Act 1998 was promulgated by the South African Parliament more than 16 years ago and emanates from the Constitution adopted in 1996. The aim of the Employment Equity Act is to achieve employment equity by (a) promoting equal opportunity and fair treatment in employment through the elimination of unfair discrimination and (b) implementing affirmative action measures in the organisation to redress employment disadvantages experienced by designated groups (Africans, Coloureds, Indians, persons with disabilities and women) to ensure their equitable representation in all occupational categories and levels in the workforce (Employment Equity Act 1998). The purpose of the Employment Equity Act is specified in section 42 which states, ‘The demographic profiles of the national and regional economically active population should be reflected in the employment areas of designated employers. This reflection will show that the workplace is redressed and equality together with a diverse and representative workforce is achieved’.

The implementation of the Employment Equity Act thus helps develop previously disadvantaged individuals and groups. However, different employees from different race groups may respond differently to such a force of change and might not be equally enthusiastic about it (Booysen 2007). This form of change might also affect employees’ level of psychological ownership. According to Yousuf (2000) some employees might experience the change as satisfactory, but for others it might bring stress, pain and disadvantages. Jordaan (2002) is of the opinion that whites experience reverse discrimination because blacks are given preference. This was confirmed in a study conducted by Janse van Rensburg and Roodt (2005), who found that blacks were more positive in terms of their perceptions of employment equity than white employees. Janse van Rensburg and Roodt (2005) also proved that perceptions of employment equity predict organisational commitment.

Pierce et al. (2001) stated that psychological ownership might lead to a number of both positive and negative effects in organisations, for example an increase in organisational commitment. No study could be found relating to the relationship between psychological ownership and perceptions of employment equity as well as the moderating role of ethnicity within this context. The question that arises is whether employees of different ethnicities experience the outcome of the Employment Equity Act as having either a positive or negative effect on their psychological ownership.

## Hypotheses

Based on the literature review and problem statement, the objective of the present study is to investigate the relationships between perceptions of employment equity and psychological ownership in a large South African mining house. This study aims to develop a structural model for predicting psychological ownership through employment equity and to determine the moderating effect of ethnicity on the relationship. It is expected that employees’ perceptions of employment equity will have a direct effect on their perceptions of psychological ownership (in terms of self-efficacy, self-identity, belongingness, accountability and territoriality). It is also expected that ethnicity (in terms of white and African participants) will moderate the effect between perceptions of employment equity and components of psychological ownership.

Given the nature of the objectives, the following hypotheses are set for this study:

### Hypothesis 1a

Perceptions of employment equity relate positively to self-efficacy, self-identity, belongingness and accountability as components of psychological ownership.

### Hypothesis 1b

Perceptions of employment equity relate negatively to territoriality as a component of psychological ownership.

### Hypothesis 2

Ethnicity moderates the effect of perceptions of employment on components of psychological ownership.

## Methods

### Research Design

A quantitative cross-sectional survey-based research design was employed to achieve the research objectives. According to Graziano and Raulin (2004) this design suits the descriptive and predictive functions associated with correlational research.

### Participants

A census-based sampling approach was employed to gather the data from a diverse group of skilled individuals employed in a large South African mining house that is considered a highly performing organisation and voted top employer within the South African context. Census-based sampling refers to a procedure of attempting to systematically acquire data from an entire population (Gupta and Kabe 2011). The respondents’ demographic information is displayed in Table 1.

**Table 1:** Respondents’ demographic information (*N* = 202)

Variable	Category	Frequency (*f*)	Percentage (%)
Gender	Male	133	65.84
	Female	69	34.16
Ethnic group	Black	58	28.71
	White	144	71.29
Age	20–29	51	25.25
	30–39	47	23.27
	40–49	60	29.70
	50+	44	21.78
Educational level	Grade 12/apprenticeship	53	26.24
	Diploma/degree	43	21.29
	Postgraduate degree	106	52.48
Operating level in organization	Operational level	50	24.75
	Junior management	51	25.25
	Middle management	67	33.17
	Senior management	34	16.83
Years working in current organization	Fewer than 5 years	70	34.65
	6–10 years	38	18.81
	11–20 years	35	17.33
	21+ years	59	29.21

The sample consisted of 65.84 % (*n* = 133) males and 34.16 % (*n* = 69) females. Of the sample, 71.29 % (*n* = 144) were white respondents and 28.71 % (*n* = 58) were African. Of the respondents, 25.25 % (*n* = 51) were 29 years and younger, 23.27 % (*n* = 47) were between 30 and 39 years of age, 29.7 % (*n* = 60) were between 40 and 49 years of age, and 21.78 % (*n* = 44) were over the age of 50. The majority of the sample had completed postgraduate studies: 52.48 % (*n* = 106). The least represented category (21.29 %; *n* = 43) was the one consisting of respondents whose highest qualification was a degree or diploma. Employees who had obtained a Grade 12 and/or an apprenticeship constituted 26.23 % (*n* = 53) of the sample. Of the respondents, 24.75 % (*n* = 50) functioned at operational level, 25.25 % (*n* = 51) at junior management level, 33.17 % (*n* = 67) at middle management level, and 14.36 % (*n* = 29) at senior management level. As indicated in Table 1, most of the sample respondents (n = 70; 34.65 %) had been working in the organisation for less than 5 years; 18.81 % (*n* = 38) had been working in the organisation between 6 and 10 years; 17.33 % (*n* = 35) had been working in the organisation between 11 and 20 years; and 29.21 % (*n* = 59) had been working in the organisation for more than 21 years.

### Measures

In order to achieve the objectives of this study, a self-developed biographic questionnaire, the Psychological Ownership Questionnaire, and an adapted version of the Perceptions of Employment Equity Questionnaire were used.

A biographical questionnaire was developed to gather biographic information on the participants relating to gender, ethnicity, age group, level of education, operational level and years employed by the organization.

The Psychological Ownership Questionnaire (POQ) developed by Avey et al. (2009) was used to measure psychological ownership. The POQ is a multi-dimensional measure consisting of 16 items; three items for each of the four components that measure promotion-oriented psychological ownership (self-efficacy, self-identity, belongingness and accountability) and four items measuring prevention-oriented psychological ownership (territoriality). The responses were captured on a six-point Likert-type scale ranging from 1 (strongly disagree) to 6 (strongly agree). Both Alberts (2012) and Olckers (2014) confirmed the construct validity of the POQ in a South African context with Cronbach’s alpha values ranging between 0.78 and 0.90 for all five dimensions.

An adapted version of the Perceptions of Employment Equity Questionnaire (EEQ), based on Martins’ theory (1999) and employed in a study by Janse van Rensburg and Roodt (2005), was used. The EEQ comprises 25 items, and responses were captured using a five-point scale ranging from 1 (to no extent) to 5 (to a very large extent). The adapted version, which comprises 16 items confirmed by Olckers (2014), produced a one factor-model of the EEQ questionnaire with a Cronbach’s alpha of 0.92.

### Research Procedure

A dual distribution channel (electronic and hardcopy) was used to distribute the questionnaires. Van Zyl and Rothmann (2012) indicated that a dual distribution channel yields higher response rates. For the electronic version, surveys were loaded on QUALTRIX for online distribution. First, electronic copies of the questionnaires were distributed via email to individuals who had access to computers. Second, where individuals did not have access to computers, a hardcopy (pen-on-paper) version of the questionnaire was distributed. Each questionnaire included a cover letter inviting subjects to participate voluntarily in the study and highlighting their rights and responsibilities. Participants were assured that their responses would remain confidential and would be used for research purposes only. Permission for the research was obtained from both the mining house and the research institution’s research ethics committee. An initial purposive sample of 411 was drawn and a sample of 202 usable questionnaires was returned, yielding a response rate of 49 %.

### Statistical Analysis

The statistical analysis was conducted with the aid of the SPSS 21 program (IBM Corporation 2012) and Mplus version 7.1 (Muthén and Muthén 2012). As part of the quantitative procedures followed, descriptive statistics (means and standard deviations) and Pearson correlations were used to describe the basic features of the data. The confidence interval of statistical significance was set at 99 % (*p* ≤ 0.01). Steyn and Swanepoel (2008) suggested effect sizes should be used as indicators of practical significance of correlations. 0.30 (medium effect) and 0.50 (large effect) were set as cut-off points for practical significance (Cohen 1988). In contrast to the traditional ‘Cronbach Alpha’ as a measure of reliability, the rho coefficients (Wang et al. 2008; Wang and Wang 2012) were used to determine the internal consistency or ‘reliability’ of the measured constructs. Rho is calculated as the proportion variance explained by a factor divided by the total variance (Wang and Wang 2012). Rothmann’s (2013) rho calculator was utilised to calculate the rho coefficient. Structural equation modelling (SEM) was employed to test four measurement models and the structural model. The maximum likelihood estimator was utilised and observed variables were classified as being measured on a continuous scale (Muthén and Muthén 2012).

In addition, the following fit indices as suggested by Hair et al. (2010) were used in the study: (a) absolute fit indices which included the Chi square statistic (which indicates absolute fit of the model), the Root-Means-Square Error of Approximation (RMSEA) and the Standardized Root Mean Residual (SRMR), and (b) incremental fit indices, including the Comparative Fit Index (CFI) and the Tucker-Lewis Index (TLI). According to Hair et al. (2010) acceptable values for the TLI and CFI should be higher than 0.90. RMSEA values lower than 0.05 and SRMR values lower than 0.08 indicate close model fit. The Wald test was employed to determine the moderation effect of ethnicity between perceptions of employment equity and psychological ownership.

Two SEM approaches were subsequently followed; first the measurement model and secondly the structural model were determined. The measurement model deals with the relationships between the measured variables and latent variables, while the structural model deals with the relationships between the latent variables only.

## Results

In order to test the hypotheses for this study, both measurement and structural models are tested and reported. First, competing measurement models are reported to determine which model proverbially ‘fits’ the data best. Secondly, the results of the structural model are reported.

### Testing Competing Measurement Models

To determine the ‘best-fitting’ measurement model, various theoretical models were systematically compared through the use of SEM. Items with poor factor loadings (≤ 0.40) were omitted (Botha and Mostert 2014). Measured items (observed variables) were used as indicators of latent variables in the measurement models (Muthén and Muthén 2012). Neither item parcelling nor correlations between error terms were permitted.

Following a confirmatory factor analysis (CFA) approach, the hypothesised measurement models were tested to determine whether the measurement items load significantly onto the scales used in the study. The observed variables were treated as continuous variables where errors of measurement were uncorrelated. Latent variables were allowed to correlate. Three items from the employment equity questionnaire (‘*Does your job allow you to make use of your abilities and talents?’; Is the remuneration fair that you receive from the organisation?’ and ‘Do you and your co*-*workers communicate openly with one another?*) were removed because of poor factor loadings (≤0.40) in order to enhance the fit of the measurement models.

The following nested measurement models were tested:

- *Model 1* A one factor model of employment equity (consisting of 13 observed variables) and five latent variables representing psychological ownership, including territoriality (4 items), self-efficacy (3 items), accountability (3 items), self-identity (3 items) and belongingness (3 items).
- *Model 2* A one factor model of employment equity (consisting of 13 observed variables) and two latent second-order variables, namely promotion-oriented psychological ownership, consisting of four latent factors, namely self-efficacy (3 items), accountability (3 items), self-identity (3 items) and belongingness (3 items), and prevention-oriented psychological ownership, consisting of one latent variable, namely territoriality (4 items).
- *Model 3* A one factor model of employment equity (consisting of 13 observed variables) and two latent second-order variables, namely promotion-oriented psychological ownership, consisting of four latent factors, namely self-efficacy (3 items), accountability (3 items), self-identity (3 items) and belongingness (3 items), and prevention-oriented psychological ownership, consisting of one latent variable, namely territoriality (4 items), loading on a third-order factor psychological ownership.
- *Model 4* A one factor model of employment equity (consisting of 13 observed variables) and one latent second-order variable psychological ownership consisting of five second order latent variables, namely self-efficacy (3 items), accountability (3 items), self-identity (3 items), belongingness (3 items) and territoriality (4 items).

Fit statistics for the competing measurement models are presented in Table 2.

**Table 2:** Fit statistics of competing measurement models

Model	χ^2^	*df*	TLI	CFI	RMSEA	SRMR	AIC	BIC
Model 1	632.81	362	0.91	0.92	0.06	0.06	13,821.91	14,160.86
Model 2	653.20	370	0.91	0.92	0.01	0.06	13,826.29	14,138.66
Model 3	662.99	371	0.91	0.92	0.01	0.07	13,834.09	14,143.13
Model 4	704.66	372	0.90	0.91	0.00	0.17	13,873.75	14,179.47

*χ* ^2^ Chi square, *df* degrees of freedom, *TLI* Tucker–Lewis Index, *CFI* Comparative Fit Index, *RMSEA* root mean square error of approximation, *SRMR* standardised root mean square residual, *AIC* Akaike information criterion, *BIC* Bayes information criterion

The Akaike Information Criterion (AIC), a comparative fit-measure, and the Bayes Information Criterion (BIC), which provides an indication of model parsimony, were used in addition to the other fit indices as indicated in Table 2. Interpreting these two fit statistics becomes meaningful when competing models are estimated. The lowest AIC value will indicate the best fitting, most parsimonious model (Muthén and Muthén 2012; Tabachnick and Fidell 2007; Wang and Wang 2012). According to the fit statistics displayed in Table 2, Model 1 fitted the data best when compared with the other models. Model 1 hypothesised that a one-factor model of employment equity (consisting of 13 observed variables) and five latent factors representing psychological ownership, including territoriality (4 items), self-efficacy (3 items), accountability (3 items), self-identity (3 items) and belongingness (3 items), fit the data the best. The observed variables in this model were treated as continuous variables and the errors of these variables were uncorrelated. This measurement model was identifiable and showed acceptable fit in relation to the competing models: χ^2^ = 632.81; *df* = 362; TLI = 0.91; CFI = 0.92; RMSEA = 0.06; SRMR = 0.06; AIC = 13,821.91; and BIC = 14,160.86.

The standardized regression coefficients in Model 1 were all significant (*p* ≤ 0.01). The β-values for employment equity ranged between 0.59 at the lowest value and 0.75 at the highest value. Territoriality ranged between 0.69 and 0.78. The highest β-value for self-efficacy was 0.93 and the lowest was 0.84. Accountability ranged from 0.71 to 0.79 while belongingness ranged from 0.85 to 0.96 and self-identity from 0.78 to 0.88.

### Testing the Structural Model

The descriptive statistics, the rho coefficients and Pearson product-moment correlations of all the measured constructs after adapting the measurement model are illustrated in Table 3. Table 3 shows that the rho coefficients (ρ) of the scales used were acceptable (ρ > 0.60) (Wang et al. 2008; Wang and Wang 2012).

**Table 3:** Descriptive statistics, rho coefficients and Pearson correlations (*N* = 202)

Variable	Mean	*SD*	*ρ*	1	2	3	4	5
1. Self-identity	4.77	1.00	0.89					
2. Belongingness	4.95	1.01	0.93	0.79**				
3. Accountability	4.67	0.89	0.80	0.48**	0.45**			
4. Self-efficacy	5.29	0.82	0.92	0.58**	0.66**	0.53**		
5. Territoriality	2.60	1.05	0.84	−0.08	−0.04	−0.04	0.02	
6. Employment equity	3.74	0.64	0.91	0.42**	0.50**	0.33**	0.40**	−0.29**

** Correlation is significant at the 0.01 level (2-tailed)

The promotion-oriented dimensions of psychological ownership, namely self-identity (r = 0.42; *p* ≤ 0.01; medium effect), belongingness (r = 0.50; *p* ≤ 0.01; large effect), accountability (r = 0.33; *p* ≤ 0.01; medium effect) and self-efficacy (r = 0.40; *p* ≤ 0.01; medium effect) all correlated significantly positively with employment equity perceptions. The prevention-oriented dimension of psychological ownership, namely territoriality (r = −0.29; *p* ≤ 0.01; small effect), correlated negatively with employment equity perceptions. All the promotion-oriented dimensions of psychological ownership correlated positively with one another.

**Fig. 1:**
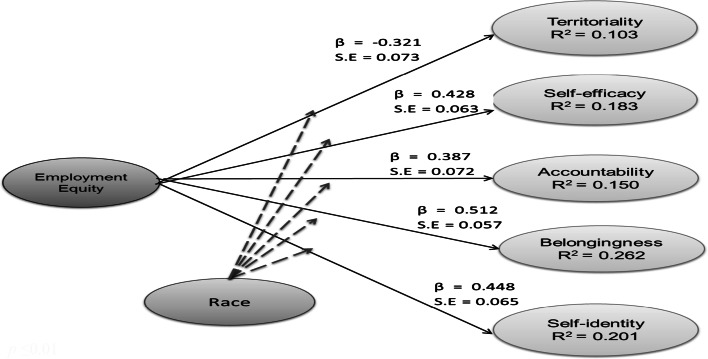
Maximum likelihood estimates for the hypothesized model. *p* ≤ 0.01

The structural model, shown in Fig. 1, was tested using measurement model 1 (c.f. Table 1) as the best fitting and most parsimonious measurement model. The structural model showed acceptable fit: χ^2^ = 632.81; *df* = 362; TLI = 0.92; CFI = 0.92; RMSEA = 0.06; and SRMR = 0.06. *Hypothesis 1a and 1b are therefore accepted.*

It was hypothesized that ethnicity (in terms of white and African) would moderate the relationship between employment equity perceptions and the components of psychological ownership. The Wald test was used to test for the differences between the two primary ethnic groups, namely the white and African group in this study. The Wald test was non-significant, suggesting that the relationship between employment equity perceptions and psychological ownership did not vary across either the white or black groups, therefore disconfirming the hypothesis that ethnicity moderates the relationship between employment equity perceptions and psychological ownership. *Hypothesis 2 is therefore not accepted.*

The results of the Wald test are displayed in Table 4. Although race did not moderate the relationship between employment equity perceptions and psychological ownership, several other observations could be made. The results revealed that employment equity perceptions were significantly positively associated with self-efficacy (white: β = 0.43, *p* ≤ 0.05; black: β = 0.80, *p* ≤ 0.01) accountability (white: β = 0.57, *p* ≤ 0.05; black: β = 0.46, *p* ≤ 0.01), belongingness (white: β = 1.04, *p* ≤ 0.05; black: β = 0.96, *p* ≤ 0.01) and self-identity (white: β = 0.94, *p* ≤ 0.05; black: β = 0.74, *p* ≤ 0.01) across racial groups and showed a significantly negative association of territory (white: β = −0.88, *p* ≤ 0.05;) with the white group. A marginal non-significant negative association with territory in the black group was produced (β = −0.39, *p* > 0.01).

**Table 4:** Wald test results

	White (N = 144)	Black (N = 59)
Β	0.21	0.50
χ^2^	634.33	709.27
Wald diff test	2.61	2.61
*df*	1	1
*p*	>0.05	>0.05

## Discussion

The objective of the present study was to investigate the relationships between perceptions of employment equity and the components of psychological ownership in a large South African mining house. A further aim was to develop a structural model for predicting psychological ownership through employment equity and to determine the moderating effect of ethnicity on the relationship. The results showed that employment equity perceptions could predict the components of psychological ownership in this sample and provided support for the structural model. Perceptions of employment equity had a direct positive effect on self-efficacy, accountability, belongingness and self-identity and a direct negative effect on territoriality in the sample. However, contrary to the original hypothesis, ethnicity did not moderate the relationship between perceptions of employment equity and psychological ownership.

Although perceptions associated with employment equity significantly affected all the components of psychological ownership, it showed the strongest relation to belongingness (27.2 % shared variance). This would indicate that perceptions of employment equity affect individuals’ perceptions of whether they belong in the organisation. If an organisation drives the implementation of legislative frameworks, such as the Employment Equity Act 1998, in order to increase the diversification of the workforce, it could impact the way in which employees relate to one another and to the organisation (Olckers 2014). Where negative perceptions exist relating to employment equity practices, employees could develop fears, resentment and alienation towards the organisation resulting in the adoption of various self-presevatory mechanisms (e.g. overt or covert sabotage) (Booysen 2007; Oosthuizen and Naidoo 2010; Yousuf 2000). As such, cognitive dissonances associated with the organisation may develop (Olckers 2014) which results in a sense of isolation and estrangement. In contrast to these findings, the implementation of employment equity practices within this organisation may have been perceived as being just and fair. Consequently, this impacted positively on employees’ sense of belongingness to the organisation as a result of the alignment between personal perceptions associated with employment equity and the manner in which these practices may have been implemented. As such, it is suggested that employment equity practices be applied fairly and consistently in order to ensure that individuals feel that they ‘have a place’ or belong within the organisation.

Similarly, the results confirmed that self-efficacy was positively affected by perceptions of employment equity. Bandura (1995) argued that various factors such as perceptions associated with one’s abilities, the complexity of work, and the situational conditions under which tasks are performed impact on self-perceived self-efficacy. Perceptions associated with employment equity, as a situational condition, could affect the way in which performance-related achievements (such as promotions and awards) are interpreted. Because fair perceptions exist relating to the implementation of employment equity practice, performance-related achievements in this organisation are attributed to the self and not to other factors such as ethnicity. These results are in line with Olckers’ findings (2014) which also showed a positive relationship between these variables.

Perceptions of employment equity also affected accountability in this study (declaring 15 % of shared variance). Accountability implies a sense of responsibility associated with one’s own actions, as well as holding others accountable for theirs (Avey et al. 2009). This implies that both employees and employers not only have to accept their responsibilities, but that they have to show voluntary transparency and answerability associated with the implementation of employment equity practice. As such, should the organisation be transparent and willing to share information/knowledge as to how it approaches and implements employment equity and the reasons thereof, a closer connection between the employee and the organisation could be established (Pierce et al. 2003). The amount of available information may provide an employee with reasons to justify the organisation’s approach and infer meaning into the reasons therefor. Employees’ may therefore more readily buy-into the process and accept its implications. Further, it could be inferred that respondents recognise the need for affirmative action to address the imbalances of the past and thus accept that some practices might promote certain race groups above others.

Self-identity was also positively predicted by perceptions of employment equity in this sample (declaring 20 % of shared variance). The development of self-identity is rooted in social constructionism, where identity is formed through the interaction with others (Avey et al. 2009) and projected through physical (e.g. car that one drives, qualifications) or meta-objects (e.g. personal perceptions or value systems) (Giddens 2013). The stronger the association with these physical or meta-objects, coupled with self-reflection on its shared meaning within the contextual boundaries, contributes to the development of self-identity (Flum 2015; Pierce et al. 2003). Perceptions of employment equity (as a meta-object) are socially constructed within the context of this organisation because individuals developed a shared meaning of the meta-object due to interactions with other employees. Therefore, perceptions of employment equity affect how employees define themselves in relation to the organisation and its values/vision/goals. As such, respondents experience shared ownership and work collaboratively to ensure that the organisation’s objectives (i.e. drive for implementing employment equity practices) are achieved.

Territoriality was negatively affected by perceptions associated with employment equity practices (declaring 10 % of shared variance). When individuals feel psychological ownership over objects, they may feel compelled to protect those objects or possessions from others by engaging in territorial behaviour (Brown et al. 2005). Territorial behaviours might include marking and defending these objects to communicate to others that employees have psychological ownership over them and to prevent others from taking or using those objects which they see as belonging exclusively to them (Brown et al. 2014). Within in this sample, should employees perceive employment equity practices and processes to be implemented unfairly it could prohibit transparency, impair collaboration/teamwork and inhibit information sharing in an attempt to forbid others from gaining control over the target of ownership (Brown et al. 2014; Olckers 2014).

The results of this study also indicated that ethnicity does not moderate the influence of perceptions of employment equity on the components of psychological ownership. This may be as a result of various contextual factors such as the professional nature of the work (skills and competent), the operational level and the maturity of the sample (as represented by the age). Research suggests that negative attitudes associated with employment equity may stem from perceptions that individuals (usually from opposite race groups, genders etc.) are employed within positions in which they are not competent nor have the required skills and abilities to perform the primary tasks but are merely employed in those positions based on their race or gender (Linkov 2014). However, given the professional nature of the respondents (74 % holding a formal qualification) the concept of competence is not questioned because they have enough skills and expertise in the work environment to be comfortable with their own abilities as well as those of their peers. As such, they are confident in their professional occupations and do not feel threatened by other ethnic groups.

Similarly, the impact of ethnicity on the relationship between perceptions of employment equity and psychological ownership can most probably be best explained through the fact that the majority of the respondents in the sample are in the age group 40–49. Respondents in this age group are normally emotionally more mature and have gained enough skills and expertise in the work environment to be comfortable with their own abilities as well as those of their peers. This is further supported by the fact that the majority of them have postgraduate degrees and as such they are confident in their professional occupations and do not feel threatened by other ethnic groups.

## Conclusions

It can be concluded that employment equity perceptions affect individuals’ self-reported sense of self-identity, self-efficacy, belongingness, accountability and territoriality. The findings highlight the importance of nurturing positive perceptions of the fairness of the implementation of employment equity practices in order to effectively predict psychological ownership. Furthermore, ethnicity did not seem to play a significant role in the prediction of psychological ownership through perceptions of employment equity.

From the results it is evident that further investigation into the relationship between employment equity perceptions and psychological ownership is required to fully understand the nuances of this intricate relationship. The relationship between the constructs and the impact of ethnicity is more complex than initially anticipated and warrants further investigation.

## Limitations and Recommendations

This study has various limitations. First, a major limitation of this study pertains to the cross-sectional research design. As a result, it was not possible to control for the use of confounding variables. Given the nature of this study, it is imperative that future research should focus on identifying causality between these constructs. It is suggested that future studies on the topic employ a longitudinal research design. Secondly, this study was limited to a single organisation and therefore has little nomothetic value. Third, the sample was not representative of the general population since only 29 % of the sample was black and data were collected at the head office of a single mining house. Common method bias was also present due to the use of self-reports. Future research should aim at qualitatively clarifying the impact of employment equity on psychological ownership and to determine the factors which impact on this relationship.
